# Common variants at 12p11, 12q24, 9p21, 9q31.2 and in *ZNF365 *are associated with breast cancer risk for *BRCA1 *and/or *BRCA2 *mutation carriers

**DOI:** 10.1186/bcr3121

**Published:** 2012-02-20

**Authors:** Antonis C Antoniou, Karoline B Kuchenbaecker, Penny Soucy, Jonathan Beesley, Xiaoqing Chen, Lesley McGuffog, Andrew Lee, Daniel Barrowdale, Sue Healey, Olga M Sinilnikova, Maria A Caligo, Niklas Loman, Katja Harbst, Annika Lindblom, Brita Arver, Richard Rosenquist, Per Karlsson, Kate Nathanson, Susan Domchek, Tim Rebbeck, Anna Jakubowska, Jan Lubinski, Katarzyna Jaworska, Katarzyna Durda, Elżbieta Złowowcka-Perłowska, Ana Osorio, Mercedes Durán, Raquel Andrés, Javier Benítez, Ute Hamann, Frans B Hogervorst, Theo A van Os, Senno Verhoef, Hanne EJ Meijers-Heijboer, Juul Wijnen, Encarna B Gómez Garcia, Marjolijn J Ligtenberg, Mieke Kriege, J Margriet Collée, Margreet GEM Ausems, Jan C Oosterwijk, Susan Peock, Debra Frost, Steve D Ellis, Radka Platte, Elena Fineberg, D Gareth Evans, Fiona Lalloo, Chris Jacobs, Ros Eeles, Julian Adlard, Rosemarie Davidson, Trevor Cole, Jackie Cook, Joan Paterson, Fiona Douglas, Carole Brewer, Shirley Hodgson, Patrick J Morrison, Lisa Walker, Mark T Rogers, Alan Donaldson, Huw Dorkins, Andrew K Godwin, Betsy Bove, Dominique Stoppa-Lyonnet, Claude Houdayer, Bruno Buecher, Antoine de Pauw, Sylvie Mazoyer, Alain Calender, Mélanie Léoné, Brigitte Bressac- de Paillerets, Olivier Caron, Hagay Sobol, Marc Frenay, Fabienne Prieur, Sandra Fert Ferrer, Isabelle Mortemousque, Saundra Buys, Mary Daly, Alexander Miron, Mary Beth Terry, John L Hopper, Esther M John, Melissa Southey, David Goldgar, Christian F Singer, Anneliese Fink-Retter, Muy-Kheng Tea, Daphne Geschwantler Kaulich, Thomas VO Hansen, Finn C Nielsen, Rosa B Barkardottir, Mia Gaudet, Tomas Kirchhoff, Vijai Joseph, Ana Dutra-Clarke, Kenneth Offit, Marion Piedmonte, Judy Kirk, David Cohn, Jean Hurteau, John Byron, James Fiorica, Amanda E Toland, Marco Montagna, Cristina Oliani, Evgeny Imyanitov, Claudine Isaacs, Laima Tihomirova, Ignacio Blanco, Conxi Lazaro, Alex Teulé, J Del Valle, Simon A Gayther, Kunle Odunsi, Jenny Gross, Beth Y Karlan, Edith Olah, Soo-Hwang Teo, Patricia A Ganz, Mary S Beattie, Cecelia M Dorfling, Elizabeth Jansen van Rensburg, Orland Diez, Ava Kwong, Rita K Schmutzler, Barbara Wappenschmidt, Christoph Engel, Alfons Meindl, Nina Ditsch, Norbert Arnold, Simone Heidemann, Dieter Niederacher, Sabine Preisler-Adams, Dorothea Gadzicki, Raymonda Varon-Mateeva, Helmut Deissler, Andrea Gehrig, Christian Sutter, Karin Kast, Britta Fiebig, Dieter Schäfer, Trinidad Caldes, Miguel de la Hoya, Heli Nevanlinna, Taru A Muranen, Bernard Lespérance, Amanda B Spurdle, Susan L Neuhausen, Yuan C Ding, Xianshu Wang, Zachary Fredericksen, Vernon S Pankratz, Noralane M Lindor, Paolo Peterlongo, Siranoush Manoukian, Bernard Peissel, Daniela Zaffaroni, Bernardo Bonanni, Loris Bernard, Riccardo Dolcetti, Laura Papi, Laura Ottini, Paolo Radice, Mark H Greene, Jennifer T Loud, Irene L Andrulis, Hilmi Ozcelik, Anna Marie Mulligan, Gord Glendon, Mads Thomassen, Anne-Marie Gerdes, Uffe B Jensen, Anne-Bine Skytte, Torben A Kruse, Georgia Chenevix-Trench, Fergus J Couch, Jacques Simard, Douglas F Easton

**Affiliations:** 1 Centre for Cancer Genetic Epidemiology, Department of Public Health and Primary Care, University of Cambridge, Worts Causeway, Cambridge CB1 8RN, UK; 2 Cancer Genomics Laboratory, Centre Hospitalier Universitaire de Québec, 2705 Laurier Boulevard, T3-57, Quebec City, QC Canada; 3 Genetics and Population Health Division, Queensland Institute of Medical Research, 300 Herston Rd, Herston, Brisbane, QLD 4006, Australia; 4 Unité Mixte de Génétique Constitutionnelle des Cancers Fréquents, Centre Hospitalier Universitaire de Lyon/Centre Léon Bérard, 28 rue Laënnec, Lyon 69373, France and INSERM U1052, CNRS UMR5286, Université Lyon 1, Cancer Research Center of Lyon, 28 rue Laënnec, Lyon 69373, France; 5 Section of Genetic Oncology, Dept. of Laboratory Medicine, University and University Hospital of Pisa, Via Roma 57, 56125 Pisa, Italy; 6 Department of Oncology, Lund University Hospital, Lund, Sweden; 7 Department of Clinical Genetics, Karolinska University Hospital, Stockholm, Sweden; 8 Department of Oncology, Karolinska University Hospital, Stockholm, Sweden; 9 Department of Genetics and Pathology, Rudbeck Laboratory, Uppsala University, Uppsala, Sweden; 10 Department of Oncology, Sahlgrenska University Hospital, Gothenburg, Sweden; 11 Abramson Cancer Center, Perelman School of Medicine at the University of Pennsylvania, Philadelphia, PA, USA; 12 Department of Genetics and Pathology, Pomeranian Medical University, Szczecin, Poland; 13 Department of Genetics and Pathology, Pomeranian Medical University, Szczecin and Postgraduate School of Molecular Medicine, Warsaw Medical University, Warsaw, Poland; 14 Human Genetics Group, Human Cancer Genetics Programme, Spanish National Cancer Research Centre, Madrid, Spain and Spanish Network on Rare Diseases (CIBERER); 15 Institute of Biology and Molecular Genetics. Universidad de Valladolid (IBGM-UVA), Valladolid, Spain; 16 Oncology unit. Hospital clinico Universitario "Lozano Blesa", Zaragoza, Spain; 17 Human Genetics Group and Genotyping Unit, Human Cancer Genetics Programme, Spanish National Cancer Research Centre, Madrid, Spain and Spanish Network on Rare Diseases (CIBERER); 18 Molecular Genetics of Breast Cancer, Deutsches Krebsforschungszentrum (DKFZ), Heidelberg, Germany; 19 Family Cancer Clinic, Netherlands Cancer Institute, Amsterdam, The Netherlands; 20 Department of Clinical Genetics, Academic Meical Center, Amsterdam, The Netherlands; 21 Department of Clinical Genetics, Netherlands Cancer Institute, Amsterdam, The Netherlands; 22 Department of Clinical Genetics, VU Medical Center, Amsterdam, The Netherlands; 23 Department of Clinical Genetics and GROM, School for Oncology and Developmental Biology, MUMC, Maastricht, The Netherlands; 24 Department of Clinical Genetics and GROM, School for Oncology and Developmental Biology, MUMC, Maastricht, The Netherlands; 25 Department of Human Genetics, Radboud University Nijmegen Medical Center, Nijmegen, The Netherlands; 26 Department of Clinical Genetics, Family Cancer Clinic, Erasmus University Medical Center, Rotterdam, The Netherlands; 27 Department of Clinical Genetics, Family Cancer Clinic, Erasmus University Medical Center, Rotterdam, The Netherlands; 28 Department of Medical Genetics, University Medical Center Utrecht, PO Box 85090, 3508 AB Utrecht, The Netherlands; 29 Department of Genetics, University Medical Center, Groningen University, Groningen, The Netherlands; 30 Netherlands Cancer Institute, Amsterdam, The Netherlands; 31 Genetic Medicine, Manchester Academic Health Sciences Centre, Central Manchester University Hospitals NHS Foundation Trust, Manchester, UK; 32 Clinical Genetics, Guy's and St. Thomas' NHS Foundation Trust, London, UK; 33 Oncogenetics Team, The Institute of Cancer Research and Royal Marsden NHS Foundation Trust, UK; 34 Yorkshire Regional Genetics Service, Leeds, UK; 35 Ferguson-Smith Centre for Clinical Genetics, Yorkhill Hospitals, Glasgow, UK; 36 West Midlands Regional Genetics Service, Birmingham Women's Hospital Healthcare NHS Trust, Edgbaston, Birmingham, UK; 37 Sheffield Clinical Genetics Service, Sheffield Children's Hospital, Sheffield, UK; 38 Department of Clinical Genetics, East Anglian Regional Genetics Service, Addenbrookes Hospital, Cambridge, UK; 39 Institute of Genetic Medicine, Centre for Life, Newcastle Upon Tyne Hospitals NHS Trust, Newcastle upon Tyne, UK; 40 Department of Clinical Genetics, Royal Devon & Exeter Hospital, Exeter, UK; 41 Medical Genetics Unit, St George's, University of London, UK; 42 Northern Ireland Regional Genetics Centre, Belfast Health and Social Care Trust, and Department of Medical Genetics, Queens University Belfast, Belfast UK; 43 Oxford Regional Genetics Service, Churchill Hospital, Oxford, UK; 44 All Wales Medical Genetics Services, University Hospital of Wales, Cardiff, UK; 45 Clinical Genetics Department, St Michael's Hospital, Bristol, UK; 46 North West Thames Regional Genetics Service, Kennedy-Galton Centre, Harrow, UK; 47 Department of Pathology and Laboratory Medicine, University of Kansas Medical Center, Kansas City, KS, USA; 48 Clinical Molecular Genetics Laboratory, Fox Chase Cancer Center, Philadelphia, PA, USA; 49 Service de Génétique Oncologique, Institut Curie, Paris, France, Unité INSERM U830, Institut Curie, Paris, France, Université Paris Descartes, Faculté de Médecine, Paris, France; 50 Service de Génétique Oncologique, Institut Curie, Paris, France and Université Paris Descartes, Faculté de Pharmacie, Paris, France; 51 Service de Génétique Oncologique, Institut Curie, 26 rue d'Ulm, Paris, France; 52 Service de Génétique Oncologique, Institut Curie, Paris, France; 53 INSERM U1052, CNRS UMR5286, Université Lyon 1, Centre de Recherche en Cancérologie de Lyon, Lyon, France; 54 Unité Mixte de Génétique Constitutionnelle des Cancers Fréquents, Hospices Civils de Lyon/Centre Léon Bérard, Lyon, France; 55 Service de Génétique, Institut de Cancérologie Gustave Roussy, Villejuif, France and INSERM U946, Fondation Jean Dausset, Paris, France; 56 Consultation de Génétique, Département de Médecine, Institut de Cancérologie Gustave Roussy, Villejuif, France; 57 Département Oncologie génétique, Prévention et Dépistage, INSERM CIC-P9502, Institut Paoli-Calmettes/Université d'Aix-Marseille II, Marseille, France; 58 Centre Antoine Lacassagne, Nice, France; 59 Service de Génétique Clinique Chromosomique et Moléculaire, Centre Hospitalier Universitaire de St Etienne, St Etienne, France; 60 Laboratoire de Génétique Chromosomique, Hôtel Dieu Centre Hospitalier, BP 1125 Chambéry, France; 61 Service de Génétique, Centre Hospitalier Universitaire Bretonneau, Tours, France; 62 Cancer Genetics Network "Groupe Génétique et Cancer", Fédération Nationale des Centres de Lutte Contre le Cancer, France; 63 Huntsman Cancer Institute, 2000 Circle of Hope, Salt Lake City, UT 84112, USA; 64 Division of Population Science, Fox Chase Cancer Center, 333 Cottman Avenue, Philadelphia, PA 19111, USA; 65 Department of Cancer Biology, Dana-Farber Cancer Institute, and Department of Surgery, Harvard Medical School, 27 Drydock Avenue, Boston, MA 02210, USA; 66 Department of Epidemiology, Columbia University, New York, NY, USA; 67 Centre for Molecular, Environmental, Genetic and Analytic (MEGA) Epidemiology, Melbourne School of Population Health, Level 1, 723 Swanston Street, The University of Melbourne, VIC 3010, Australia; 68 Department of Epidemiology, Cancer Prevention Institute of California, 2201 Walnut Avenue, Suite 300, Fremont, CA 94538, USA; 69 Genetic Epidemiology Laboratory, Department of Pathology, University of Melbourne, Australia; 70 Department of Dermatology, University of Utah School of Medicine, 30 North 1900 East, SOM 4B454, Salt Lake City, UT 84132, USA; 71 Dept of OB/GYN and Comprehensive Cancer Center, Medical University of Vienna, Vienna, Austria; 72 Center for Genomic Medicine, Rigshospitalet, Copenhagen University Hospital, Copenhagen, Denmark; 73 Department of Pathology, Landspitali - University Hospital, Reykjavik Iceland and Faculty of Medicine, University of Iceland, Reykjavik, Iceland; 74 Epidemiology Research Program, American Cancer Society, Atlanta, GA, USA; 75 Department of Environmental Medicine, NYU Cancer Institute, New York University School of Medicine, New York, NY, USA; 76 Clinical Cancer Genetics Laboratory, Memorial Sloane Kettering Cancer Center, New York, NY, USA; 77 Statistical and Data Center, Roswell Park Cancer Institute, Buffalo, NY, USA; 78 Australia New Zealand (ANZGOG), Westmead Hospital, Sydney, Australia; 79 Ohio State University, Columbus Cancer Council, Columbus, OH, USA; 80 Evanston CCOP - NorthShore University Health System; University of Chicago, Chicago, IL, USA; 81 Southern Pines Women's Health Center, P.C., University of North Carolina at Chapel Hill, Chapel Hill, NC, USA; 82 Sarasota Memorial Healthcare, Tufts Medical Center, Sarasota, Florida, USA; 83 Department of Molecular Virology, Immunology and Medical Genetics and Internal Medicine, Comprehensive Cancer Center, The Ohio State University, Columbus, OH 43210, USA; 84 Immunology and Molecular Oncology Unit, Istituto Oncologico Veneto IOV - IRCCS, Padua, Italy; 85 U.O.C. di Oncologia, ULSS5 Ovest Vicentino, Italy; 86 Laboratory of Molecular Oncology, N.N. Petrov Institute of Oncology, St.-Petersburg, Russia; 87 Lombardi Comprehensive Cancer Center, Georgetown University, Washington DC, USA; 88 Latvian Biomedical Research and Study Centre, Latvia; 89 Genetic Counselling Unit, Hereditary Cancer Program, IDIBELL-Catalan Institute of Oncology, Barcelona, Spain; 90 Molecular Diagnostic Unit, Hereditary Cancer Program, IDIBELL-Catalan Institute of Oncology, Barcelona, Spain; 91 Department of Preventive Medicine, Keck School of Medicine, University of Southern California, Los Angeles, CA, USA; 92 Department of Gynecologic Oncology, Roswell Park Cancer Institute, Buffalo, NY, USA; 93 Women's Cancer Program at the Samuel Oschin Comprehensive Cancer Institute at Cedars-Sinai Medical Center, Los Angeles, CA, USA; 94 Department of Molecular Genetics, National Institute of Oncology, Budapest, Hungary; 95 Cancer Research Initiatives Foundation, Sime Darby Medical Centre, Malaysia and University Malaya Cancer Research Institute, University Malaya Medical Centre, Kuala Lumpur, Malaysia; 96 Jonsson Comprehensive Cancer Center at UCLA, Los Angeles, CA, USA; 97 UCSF Cancer Risk Program, University of California, San Francisco, CA; UCSF Departments of Medicine, Epidemiology, and Biostatistics, Sand Francisco, CA, USA; 98 Cancer Genetics Laboratory, Department of Genetics, University of Pretoria, South Africa; 99 Oncogenetics Laboratory. Vall d'Hebron Institute of Oncology (VHIO), Vall d'Hebron University Hospital. Barcelona, Spain; 100 The Hong Kong Hereditary Breast Cancer Family Registry; The Universtiy of Hong Kong; Cancer Genetics Center, Hong Kong Sanatorium and Hospital, Hong Kong; 101 Centre of Familial Breast and Ovarian Cancer, Department of Gynaecology and Obstetrics and Centre for Integrated Oncology (CIO), University hospital of Cologne, Cologne, Germany; 102 Institute for Medical Informatics, Statistics and Epidemiology, University of Leipzig, Leipzig, Germany; 103 Department of Gynaecology and Obstetrics, Division of Tumour Genetics, Klinikum rechts der Isar, Technical University Munich, Munich, Germany; 104 Department of Gynaecology and Obstetrics, Ludwig-Maximilian University Munich, Munich, Germany; 105 Department of Gynaecology and Obstetrics, University Hospital of Schleswig-Holstein, Campus Kiel, Christian-Albrechts University Kiel, Kiel, Germany; 106 Institute of Human Genetics, University Hospital of Schleswig-Holstein, Campus Kiel, Christian-Albrechts University Kiel, Kiel, Germany; 107 Department of Gynaecology and Obstetrics, University Hospital Düsseldorf, Heinrich-Heine University, Düsseldorf, Germany; 108 Institute of Human Genetics, University of Münster, Münster, Germany; 109 Institute of Cell and Molecular Pathology, Hannover Medical School, Hannover, Germany; 110 Institute of Human Genetics, Campus Virchov Klinikum, Charite Berlin, Germany; 111 Department of Gynaecology and Obstetrics, University Hospital Ulm, Germany; 112 Centre of Familial Breast and Ovarian Cancer, Department of Medical Genetics, Institute of Human Genetics, University Würzburg, Würzburg, Germany; 113 Institute of Human Genetics, Department of Human Genetics, University Hospital Heidelberg, Germany; 114 Department of Gynaecology and Obstetrics, University Hospital Carl Gustav Carus, Technical University. Dresden, Germany; 115 Institute of Human Genetics, University Regensburg, Regensbirg. Germany; 116 Institute of Human Genetics, University Hospital Frankfurt a.M., Germany Molecular Oncology Laboratory, Hospital Clinico San Carlos, Madrid, Spain; 117 Molecular Oncology Laboratory, Hospital Clinico San Carlos, Martin Lagos s/n, Madrid, Spain; 118 Department of Obstetrics and Gynecology, University of Helsinki and Helsinki University Central Hospital, Biomedicum Helsinki, P.O. BOX 700, 00029 HUS, Helsinki, Finland; 119 Faculty of Medicine - Medicine and Medical Specialties, Université de Montréal Hemato-oncology service, Hôpital du Sacré-Coeur de Montréal, 5400 Gouin Blvd West Montreal, QC, Canada; 120 Peter MacCallum Cancer Center, Melbourne, VIC, Australia; 121 Department of Population Sciences, Beckman Research Institute of City of Hope, Duarte, CA, USA; 122 Department of Laboratory Medicine and Pathology, Mayo Clinic, Rochester, MN, USA; 123 Department of Medical Genetics, Mayo Clinic, Rochester, MN, USA; 124 Department of Health Sciences Research, Mayo Clinic, Rochester, MN, USA; 125 Unit of Molecular Bases of Genetic Risk and Genetic Testing, Department of Preventive and Predicted Medicine, Fondazione IRCCS Istituto Nazionale Tumouri (INT), Milan, Italy and IFOM, Fondazione Istituto FIRC di Oncologia Molecolare, Milan, Italy; 126 Unit of Medical Genetics, Department of Preventive and Predictive Medicine, Fondazione IRCCS Istituto Nazionale Tumouri (INT), Milan, Italy; 127 Division of Cancer Prevention and Genetics, Istituto Europeo di Oncologia (IEO), Milan Italy; 128 Department of Experimental Oncology, Istituto Europeo di Oncologia, Milan, Italy and Consortium for Genomics Technology (Cogentech), Milan, Italy; 129 Cancer Bioimmunotherapy Unit, Centro di Riferimento Oncologico, IRCCS, Aviano (PN), Italy; 130 Medical Genetics Unit, Department of Clinical Physiopathology, University of Florence, Firenze, Italy; 131 Department of Molecular Medicine, "Sapienza" University of Rome, Rome, Italy; 132 Clinical Genetics Branch, DCEG, NCI; Room EPS 7032, Rockville, MD 20852, USA; 133 Samuel Lunenfeld Research Institute, Mount Sinai Hospital, Toronto, ON; Cancer Care Ontario, Departments of Molecular Genetics and Laboratory Medicine and Pathobiology, University of Toronto, ON, Canada; 134 Samuel Lunenfeld Research Institute, Mount Sinai Hospital, Toronto, ON; Department of Laboratory Medicine and Pathobiology, University of Toronto, ON, Canada; 135 Department of Laboratory Medicine and Pathobiology, University of Toronto, Toronto, ON, Canada; Department of Laboratory Medicine, and the Keenan Research Centre of the Li Ka Shing Knowledge Institute, St Michael's Hospital, Toronto, ON, Canada; 136 Ontario Cancer Genetics Network: Cancer Care Ontario, Toronto, ON, Canada; 137 Department of Clinical Genetics, Odense University Hospital, Denmark; 138 Department of Clincial Genetics, Rigshospital and Copenhagen University, Denmark; 139 Department of Clinical Genetics, Skejby Hospital, Aarhus, Denmark; 140 Department of Clinical Genetics, Vejle Hospital, Denmark; 141 Department of Laboratory Medicine and Pathology, and Health Sciences Research, Mayo Clinic, Rochester, MN, USA; 142 Cancer Genomics Laboratory, Centre Hospitalier Universitaire de Québec, 2705 Laurier Boulevard, T3-57, Quebec City and Canada Research Chair in Oncogenetics, Department of Molecular Medicine, Faculty of Medicine, Laval University, QC, Canada

## Abstract

**Introduction:**

Several common alleles have been shown to be associated with breast and/or ovarian cancer risk for *BRCA1 *and *BRCA2 *mutation carriers. Recent genome-wide association studies of breast cancer have identified eight additional breast cancer susceptibility loci: rs1011970 (9p21, *CDKN2A/B)*, rs10995190 (*ZNF365)*, rs704010 (*ZMIZ1*), rs2380205 (10p15), rs614367 (11q13), rs1292011 (12q24), rs10771399 (12p11 near *PTHLH) *and rs865686 (9q31.2).

**Methods:**

To evaluate whether these single nucleotide polymorphisms (SNPs) are associated with breast cancer risk for *BRCA1 *and *BRCA2 *carriers, we genotyped these SNPs in 12,599 *BRCA1 *and 7,132 *BRCA2 *mutation carriers and analysed the associations with breast cancer risk within a retrospective likelihood framework.

**Results:**

Only SNP rs10771399 near *PTHLH *was associated with breast cancer risk for *BRCA1 *mutation carriers (per-allele hazard ratio (HR) = 0.87, 95% CI: 0.81 to 0.94, *P*-trend = 3 × 10^-4^). The association was restricted to mutations proven or predicted to lead to absence of protein expression (HR = 0.82, 95% CI: 0.74 to 0.90, *P*-trend = 3.1 × 10^-5^, *P*-difference = 0.03). Four SNPs were associated with the risk of breast cancer for *BRCA2 *mutation carriers: rs10995190, *P*-trend = 0.015; rs1011970, *P*-trend = 0.048; rs865686, 2df-*P *= 0.007; rs1292011 2df-*P *= 0.03. rs10771399 (*PTHLH*) was predominantly associated with estrogen receptor (ER)-negative breast cancer for *BRCA1 *mutation carriers (HR = 0.81, 95% CI: 0.74 to 0.90, *P*-trend = 4 × 10^-5^) and there was marginal evidence of association with ER-negative breast cancer for *BRCA2 *mutation carriers (HR = 0.78, 95% CI: 0.62 to 1.00, *P*-trend = 0.049).

**Conclusions:**

The present findings, in combination with previously identified modifiers of risk, will ultimately lead to more accurate risk prediction and an improved understanding of the disease etiology in *BRCA1 *and *BRCA2 *mutation carriers.

## Introduction

Pathogenic mutations in *BRCA1 *and *BRCA2 *confer high risks of breast and ovarian cancers [1,2]. Several lines of evidence suggest that these risks are modified by other genetic or environmental factors that cluster in families. Direct evidence for genetic modifiers of risk has been provided through studies that investigated the associations between common breast and ovarian cancer susceptibility variants, identified through genome-wide association studies (GWAS) or candidate gene studies in the general population, and cancer risk for *BRCA1 *and *BRCA2 *mutation carriers [3-8] and through GWAS in *BRCA1 *and *BRCA2 *mutation carriers [9-11]. Six loci (at *TOX3*, 2q35, 6q25.1, 19p13, *CASP8 *and wild-type copy of *BRCA1*) are now known to be associated with breast cancer risk for *BRCA1 *mutation carriers; a further 10 loci (at *FGFR2, TOX3, MAP3K1, LSP1*, 2q35, *SLC4A7*, 5p12, 1p11.2, *ZNF365 *and *RAD51*) have been associated with breast cancer risk for *BRCA2 *carriers. The association patterns between these common variants and breast cancer risk for *BRCA1 *and *BRCA2 *mutation carriers are in general different, and mostly reflect differences in the associations of these single-nucleotide polymorphism (SNPs) with estrogen receptor (ER) status of breast cancer [12-14].

GWAS in the general population have recently identified eight additional breast cancer susceptibility loci which have not been previously investigated in *BRCA1 *and *BRCA2 *mutation carriers. Turnbull *et al. *[15] identified five susceptibility loci on chromosomes 9 (rs1011970), 10 (rs2380205, rs10995190, rs704010) and 11 (rs614367) through a GWAS of breast cancer cases with a family history of the disease and unrelated controls. In a further follow-up of additional promising associations from that GWAS, the Breast Cancer Association Consortium (BCAC) has identified two additional loci at 12p11 (rs10771399) and 12q24 (rs1292011) which were associated with breast cancer risk in the general population [16]. The estimated odds ratios (OR) for ER-positive breast cancer for four of these SNPs (rs1011970 near *CDKN2A/CDKN2B *at chromosome 9, rs10995190 in *ZNF365 *at chromosome 10, rs614367 at 11q13 and rs1292011 at 12q24) were higher than the OR estimates for ER-negative breast cancer. In contrast, the OR estimates were similar for ER-positive and ER-negative breast cancer for SNPs rs2380205 (near *ANKRD16 *and *FBXO18*), rs704010 (upstream of *ZMIZ1*) and rs10771399 near *PTHLH*. In a separate GWAS that included mainly cases with two primary breast cancers or a family history of the disease, SNP rs865686 at 9q31.2 was found to be associated with risk for breast cancer, OR = 0.89 (95% CI: 0.85 to 0.92), but no estimates by ER status were reported [17].

The associations of these eight loci with breast cancer risk for *BRCA1 *and *BRCA2 *mutation carriers are still unknown. To evaluate these associations, we genotyped the eight SNPs in *BRCA1 *and *BRCA2 *mutation carriers participating in the Consortium of Investigators of Modifiers of *BRCA1/2 *(CIMBA). We further investigated the associations with the risks of developing ER-positive and ER-negative breast cancer and the risk of ovarian cancer.

## Materials and methods

### Subjects

All carriers participated in clinical or research studies at the host institutions which have been approved by local ethics committees (list provided in Additional file 1 Table S1). Informed consent was obtained from all study participants. Subjects were *BRCA1 *and *BRCA2 *mutation carriers recruited by 40 study centres in 28 countries through CIMBA (Additional file 1 Table 2). The majority of carriers (97.58%) were recruited through cancer genetics clinics offering genetic testing, and enrolled into national or regional studies. Some carriers were identified by population-based sampling of cases (2.38%), and some by community recruitment (0.04%). Eligibility to participate in CIMBA is restricted to female carriers of pathogenic *BRCA1 *or *BRCA2 *mutations age 18 years old or older at recruitment. Information collected included the year of birth; mutation description, including nucleotide position and base change; self reported ethnic ancestry, age at last follow-up; ages at breast or ovarian cancer diagnoses; and age or date at bilateral prophylactic mastectomy and oophorectomy. Related individuals were identified through a unique family identifier. Women were included in the analysis if they carried mutations that were pathogenic according to generally recognized criteria [18]. Further details on CIMBA can be found elsewhere [19].

Women who carried pathogenic mutations in both *BRCA1 *and *BRCA2 *were excluded from the current analysis. The primary analysis was restricted to women self-reported as "white of European ancestry". The number of mutation carriers of non-white ancestry was too small to allow separate analysis. We investigated possible overlap of carriers between studies by comparing the year of birth, exact mutation description and the reported ages, to identify potential duplicate individuals. Where possible we also used other genotype data on SNPs genotyped in the current round (at least 26 SNPs), in previous genotyping rounds or as part of GWAS to find hidden duplicates. When a potential duplicate was identified, we contacted the relevant centres for further information about these individuals, in a manner that protected the identity of the individuals in question, in order to determine precisely the extent of true overlap in subjects and families appearing more than once in the data set. Duplicate mutation carriers were included only once in the analysis. When in doubt, and when centres could not clarify a potential duplication, one of the samples was excluded from the analysis.

### Genotyping

DNA samples (in almost all cases, obtained from blood) were genotyped using the iPLEX Mass Array platform at four genotyping centres (Additional file 1 Table S2); the iPLEX included 26 SNPs as part of a larger study. All centres included at least 2% of the samples in duplicate, no template controls in every plate, and a random mixture of affected and unaffected carriers. Samples that failed for ≥ 20% of all the SNPs typed (that is, five or more) were excluded from the analysis. A study was included in the analysis only if the call rate was over 95%, after samples that failed at multiple SNPs had been excluded. For each study, genotypes for at least 98% of the duplicate samples had to be concordant. To assess the accuracy of genotyping across genotyping centres, the four centres genotyped 95 DNA samples from a standard test plate (Coriell Institute) for all SNPs. If the genotyping was inconsistent for more than one sample in the test plate, all the studies genotyped at the centre were excluded from the analysis of that SNP. No SNPs failed this criterion. The present study included eight SNPs: rs1011970 (9p21, near *CDKN2A/B*), rs10995190 (10q21, near *ZNF365*), rs704010 (10q22, near ZMIZ1), rs2380205 (10p15), rs614367 (11q13), rs1292011 (12q24), rs10771399 (12p11 near *PTHLH*) and rs865686 (9q31.2). Based on the quality control criteria, 4 studies were excluded from the analysis of rs2380205 (one due to low duplicate concordance, 3 due to low call rate), 2 studies were excluded from the analysis of rs704010 (low call rate) and 13 studies were excluded from the analysis of rs1292011 (all due to low call rates). As an additional genotyping quality-control check, we also evaluated the deviation from Hardy-Weinberg equilibrium (HWE) for unrelated subjects separately for each SNP and study. Nine studies had HWE *P*-values in the range 0.005 to 0.05 (two studies for rs10995190, two studies for rs704010, one study for rs10771399, two for rs1292011 and two for rs865686). Upon examination of the cluster plots for these studies and SNPs, none revealed any unusual patterns and these studies were included in all the analyses. After the above exclusions, a total of 19,731 unique mutation carriers (12,599 *BRCA1 *and 7,132 *BRCA2*) from 40 studies had an observed genotype for at least 1 of the SNPs and were included in the primary analysis.

### Statistical analysis

The aim of the primary analysis was to evaluate the association between each genotype and breast cancer risk within a survival analysis framework. The time variable for each individual was defined to be the time to breast cancer diagnosis. Each individual was followed until the first breast cancer diagnosis, ovarian cancer diagnosis, or bilateral prophylactic mastectomy or the age at last observation. Only those with a first breast cancer diagnosis were considered as affected in the analysis. Mutation carriers censored at ovarian cancer diagnosis were considered unaffected. Analysis was conducted by modelling the retrospective likelihood of the observed genotypes conditional on the disease phenotypes as previously described [18]. The effect of each SNP was modelled either as a per-allele hazard ratio (HR) (multiplicative model) or as separate HRs for heterozygotes and homozygotes, and these were estimated on the logarithmic scale. The HRs were assumed to be independent of age (that is, we used a Cox proportional-hazards model). The assumption of proportional hazards was tested by adding a "genotype x age" interaction term to the model in order to fit models in which the HR changed with age. Analyses were carried out with the pedigree-analysis software MENDEL [20]; details of this approach have been described previously [18,21]. We examined between-study heterogeneity by comparing the models that allowed for study-specific log-hazard ratios against models in which the same log-hazard ratio was assumed to apply to all studies.

To investigate whether our results were influenced by any of our assumptions we performed additional sensitivity analyses. If a SNP is associated with disease survival, the inclusion of prevalent cases may influence the HR estimates. Current data indicate that five-year survival after a breast cancer diagnosis is over 80% (Cancer Research - UK, Breast cancer survival statistics) and studies have suggested no difference in survival between mutation carriers and non-carriers [22]. We, therefore, repeated our analysis by excluding mutation carriers diagnosed more than five years prior to recruitment into the study. To examine whether SNP associations differed by type of mutation, we classified *BRCA1 *mutations according to their potential functional effect [23-26]. Class 1 mutations were those likely to lead to the absence of protein expression due to i) reduced transcript level and/or degradation or instability of truncated proteins, or ii) absence of transcription. Class 1 mutations comprise truncating mutations expected to trigger nonsense-mediated mRNA decay (NMD) or translation re-initiation but no production of stable protein, and deletion of transcription regulatory regions. Class 2 mutations were those likely to generate stable mutant proteins with partial or total loss of function that might also have dominant negative effect. Class 2 mutations include missense substitutions, in-frame deletions and insertions, as well as truncating mutations with premature stop codons occurring in the last exon. Mutations whose consequences at transcript or protein level could not be inferred were not considered for this classification. These were mainly mutations located in splice sites but not characterised for their effect at the transcript level, or large deletions or insertions with undetermined boundaries.

The associations of these SNPs with ovarian cancer risk were evaluated within a competing risk analysis framework [8,9,21], by estimating HRs simultaneously for breast and ovarian cancers. In this model, each individual was at risk of developing either breast or ovarian cancer, by assuming that the probabilities of developing each disease were independent conditional on the underlying genotype. A different censoring process was used for the competing risk analysis, whereby individuals were followed up to the age of the first breast or ovarian cancer diagnosis and were considered to have developed the corresponding disease. No follow-up was considered after the first cancer diagnosis. Individuals were censored for breast cancer at the age of bilateral prophylactic mastectomy and for ovarian cancer at the age of bilateral oophorectomy and were assumed to be unaffected for the corresponding disease. The remaining individuals were censored at the age at last observation and were assumed to be unaffected for both diseases.

We further evaluated the associations of these SNPs with breast cancer subtypes defined by the estrogen receptor (ER) status of the tumours in *BRCA1 *and *BRCA2 *mutation carriers. The analysis was carried out by an extension of the retrospective likelihood approach to model the simultaneous effect of each SNP on more than one tumour subtype [14]. Briefly, this involves modelling the conditional likelihood of the observed SNP genotypes and tumour subtypes, given the disease phenotypes. Within this framework it is possible to estimate simultaneously the HRs for each tumour subtype and test for heterogeneity in the associations. Only studies that provided tumour pathology information and had genotype information were included in the analysis. To maximise the available information, genotyped mutation carriers that were missing information on tumour characteristics (within each study) were included in the analysis, and their disease subtype was assumed to be missing at random [14]. This is a reasonable assumption given that more than 90% of mutation carriers in our sample were recruited prior to 2007, when it was uncommon to use tumour pathology in selecting individuals for *BRCA1 *and *BRCA2 *mutation screening.

To ensure a sufficiently large number of mutation carriers within each stratum, we grouped studies from the same country. All analyses were stratified by country of residence and used calendar-year- and cohort-specific cancer incidences for *BRCA1 *and *BRCA2 *[27]. For sensitivity analyses, strata with small numbers of mutation carriers were grouped. We used a robust variance-estimation approach to allow for the non-independence among related carriers [28].

## Results

The analysis included 12,599 *BRCA1 *and 7,132 *BRCA2 *mutation carriers who were genotyped successfully for at least one of the eight SNPs. Table 1 summarises the characteristics of the mutation carriers used in the analysis. In evaluating associations with breast cancer, 10,200 mutation carriers had been diagnosed with a first breast cancer diagnosis, 1,869 were censored at an ovarian cancer diagnosis, 561 at age of bilateral prophylactic mastectomy and 7,101 at the age at last observation.

**Table 1:** Summary characteristics for the 19731 eligible *BRCA1 *and *BRCA2 *carriers* used in the analysis

Characteristic	*BRCA1*		*BRCA2*	
	Unaffected	Breast cancer	Unaffected	Breast cancer
Number	6,209	6,390	3,322	3,810
Person-years follow-up	264,903	263,068	147,053	168,201
Median age at censure (IQR^1^)	42 (34 to 50)	40 (34 to 47)	43 (34 to 53)	43 (37 to 50)
Age at censure, N (%)				
< 30	1,189 (19.2)	691 (10.8)	611 (18.4)	306 (8.0)
30 to 39	1,161 (26.8)	2,445 (38.3)	834 (25.1)	1,141 (30.0)
40 to 49	1,765 (28.4)	2,191 (34.3)	865 (26.0)	1,394 (36.6)
50 to 59	1,058 (17.0)	812 (12.7)	566 (17.0)	687 (18.0)
60 to 69	380 (6.1)	198 (3.1)	302 (9.1)	226 (5.9)
70+	156 (2.5)	53 (0.8)	144 (4.3)	56 (1.5)
Year of birth, N (%)				
< 1920	28 (0.5)	30 (0.5)	23 (0.7)	44 (1.2)
1920 to 1929	131 (2.1)	196 (3.1)	99 (3.0)	167 (4.4)
1930 to 1939	369 (5.9)	516 (8.1)	232 (7.0)	430 (11.3)
1940 to 1949	832 (13.4)	1,341 (21.0)	458 (13.8)	896 (23.5)
1950 to 1959	1,409 (22.7)	1,989 (31.1)	691 (20.8)	1,160 (30.5)
1960 to 1969	1,703 (27.4)	1,666 (26.1)	902 (27.2)	868 (22.8)
1970+	1,737 (28.0)	652 (10.2)	917 (27.6)	245 (6.4)
Mutation Class, N (%)				
Class 1^2^	4,063 (65.4)	3,878 (60.7)	3,114 (93.7)	3,520 (92.4)
Class 2^2^	1,780 (28.7)	1,973 (30.9)	72 (2.2)	100 (2.6)
Other	366 (5.9)	539 (8.4)	136 (4.1)	190 (5.0)

^1 ^IQR: Interquartile Range

^2 ^See methods for definitions

* Carriers of self-reported white European ancestry only.

### Associations with cancer risk for BRCA1 mutation carriers

Of the eight SNPs, only rs10771399 in *PTHLH *was associated with breast cancer risk for *BRCA1 *mutation carriers (*P*-trend = 3 × 10^-4^, Table 2). The association was consistent with a multiplicative model in which each copy of the minor allele was estimated to confer a HR of 0.87 (95% CI: 0.81 to 0.94). There was no evidence of heterogeneity in the HR estimates across studies (*P*-het = 0.24, Additional file 1 Supplementary Figure 1). There was no evidence that the HRs varied with age (*P *= 0.68). The association remained significant, with a similar HR estimate (HR = 0.85, 95% CI: 0.77 to 0.93, *P*-trend = 6 × 10^-4^, Table 3), when long-term survivors were excluded from the analysis, suggesting no evidence of survival bias. Interestingly, the association was restricted to *BRCA1 *carriers of Class 1 mutations (HR = 0.82, 95% CI: 0.74 to 0.90, *P*-trend = 3 × 10^-5^, Table 3) with no evidence of association for Class 2 mutation carriers (HR = 1.00, 0.87 to 1.15, *P*-trend = 0.99, *P*-difference between Class 1 and Class 2 = 0.03).

**Table 2:** SNP genotype distributions and associations with breast cancer risk.

Mutation	Genotype	Unaffected N (%)	Affected^a^ N (%)	HR	95% CI	*P*-value
**CDK2NA/B - rs1011970**						
*BRCA1*	GG	4,318 (69.7)	4,460 (70.0)	1		
	GT	1,698 (27.4)	1,719 (27.0)	1.01	0.94 to 1.09	
	TT	180 (2.9)	195 (3.1)	1.11	0.91 to 1.35	
	2-df test					0.61
	per allele			1.03	0.96 to 1.09	0.45
*BRCA2*	GG	2,279 (68.7)	2,586 (67.9)	1		
	GT	943 (28.4)	1,098 (28.9)	1.08	0.98 to 1.19	
	TT	94 (2.8)	123 (3.2)	1.23	0.95 to 1.59	
	2-df test					0.12
	per allele			**1.09**	**1.00 to 1.18**	**0.048**
**ZNF365 - rs10995190**						
*BRCA1*	GG	4,394 (70.9)	4,556 (71.5)	1		
	GA	1,656 (26.7)	1,662 (26.1)	0.98	0.91 to 1.06	
	AA	147 (2.4)	156 (2.5)	0.98	0.79 to 1.20	
	2-df test					0.89
	per allele			0.99	0.93 to 1.05	0.64
*BRCA2*	GG	2,334 (70.4)	2,802 (73.7)	1		
	GA	913 (27.5)	923 (24.3)	**0.86**	**0.78 to 0.96**	
	AA	68 (20.1)	79 (2.1)	0.96	0.69 to 1.34	
	2-df test					**0.019**
	per allele			**0.90**	**0.82 to 0.98**	**0.015**
**ZMIZ1 - rs704010**						
*BRCA1*	CC	2,476 (40.3)	2,504 (39.8)	1		
	CT	2,814 (45.8)	2,894 (46.0)	1.03	0.96 to 1.10	
	TT	855 (13.9)	888 (14.1)	1.04	0.93 to 1.15	
	2-df test					0.69
	per allele			1.02	0.97 to 1.07	0.42
*BRCA2*	CC	1,286 (39.3)	1,443 (38.4)	1		
	CT	1,496 (45.7)	1,779 (47.3)	1.07	0.97 to 1.18	
	TT	494 (15.8)	539 (14.3)	0.99	0.86 to 1.14	
	2-df test					0.32
	per allele			1.01	0.95 to 1.08	0.73
**10p15 - rs2380205**						
*BRCA1*	CC	1,609 (32.5)	1,710 (32.1)	1		
	CT	2,410 (48.7)	2,625 (49.3)	1.01	0.93 to 1.09	
	TT	933 (18.8)	990 (18.6)	1.02	0.92 to 1.13	
	2-df test					0.95
	per allele			1.01	0.96 to 1.06	0.75
*BRCA2*	CC	1,013 (32.8)	1,163 (31.8)	1		
	CT	1,516 (49.1)	1,816 (49.7)	1.05	0.95 to 1.16	
	TT	560 (18.1)	681 (18.6)	1.03	0.90 to 1.17	
	2-df test					0.63
	per allele			1.02	0.96 to 1.09	0.57
**11q13 - rs614367**						
*BRCA1*	CC	4,516 (73.2)	4,581 (72.1)	1		
	CT	1,511 (24.5)	1,618 (25.5)	1.05	0.98 to 1.14	
	TT	146 (2.4)	154 (2.4)	1.07	0.87 to 1.32	
	2-df test					0.34
	per allele			1.05	0.98 to 1.12	0.15
*BRCA2*	CC	2,432 (73.6)	2,723 (71.8)	1		
	CT	799 (24.1)	983 (26.0)	1.06	0.96 to 1.17	
	TT	76 (2.3)	83 (2.2)	0.97	0.72 to 1.30	
	2-df test					0.54
	per allele			1.03	0.95 to 1.12	0.46
**12q24 - rs1292011**						
*BRCA1*	AA	1,292 (34.3)	1,331 (35.4)	1		
	AG	1,825 (48.4)	1,775 (47.3)	0.98	0.89 to 1.07	
	GG	653 (17.3)	649 (17.3)	1.01	0.90 to 1.14	
	2-df test					0.80
	per allele			1.00	0.94 to 1.06	0.99
*BRCA2*	AA	824 (35.2)	908 (35.9)	1		
	AG	1,095 (46.7)	1,225 (48.4)	1.03	0.92 to 1.16	
	GG	423 (18.1)	397 (15.7)	**0.84**	**0.72 to 0.99**	
	2-df test					**0.03**
	per allele			0.94	0.87 to 1.01	0.10
**PTHLH - rs10771399**						
*BRCA1*	AA	4,913 (79.4)	5,221 (82.0)	**1**		
	AG	1,194 (19.3)	1,082 (17.0)	**0.87**	**0.80 to 0.95**	
	GG	83 (1.3)	65 (1.0)	**0.77**	**0.57 to 1.04**	
	2-df test					**1.5 × 10^-3^**
	per allele			**0.87**	**0.81 to 0.94**	**3.2 × 10^-4^**
*BRCA2*	AA	2,649 (80.0)	3,085 (81.2)	1		
	AG	620 (18.7)	679 (17.9)	0.95	0.85 to 1.07	
	GG	45 (1.4)	34 (0.9)	0.74	0.47 to 1.15	
	2-df test					0.31
	per allele			0.93	0.84 to 1.04	0.20
**9q31.2 - rs865686**						
*BRCA1*	TT	2,521 (40.1)	2,640 (41.4)	1		
	TG	2,872 (46.4)	2,849 (44.7)	0.95	0.88 to 1.01	
	GG	799 (12.9)	880 (13.8)	1.05	0.95 to 1.17	
	2-df test					0.06
	per allele			1	0.96 to 1.05	0.85
*BRCA2*	TT	1,277 (38.6)	1,581 (41.6)	1		
	TG	1,610 (48.6)	1,717 (45.2)	**0.86**	**0.78 to 0.95**	
	GG	425 (12.8)	501 (13.2)	0.96	0.84 to 1.11	
	2-df test					**7.3 × 10^-3^**
	per allele			0.95	0.89 to 1.01	0.10

^a ^Breast Cancer

HR, hazard ratio

**Table 3:** Associations with breast cancer risk, after excluding prevalent breast cancer cases, and *BRCA1 *mutation class.

	Unaffected, N	Affected, N	HR	95% CI	*P*-value
**Excluding prevalent breast cancer cases**					
**CDK2NA/B -rs1011970**					
*BRCA1*	6,200	3,152	1.05	0.98 to 1.14	0.18
*BRCA2*	3,319	1,950	1.10	1.00 to 1.22	0.05
**ZNF365 - rs10995190**					
*BRCA1*	6,201	3,151	0.96	0.89 to 1.04	0.34
*BRCA2*	3,318	1,949	0.90	0.81 to 1.00	0.05
**ZMIZ1 - rs704010**					
*BRCA1*	6,149	3,094	1.02	0.96 to 1.08	0.53
*BRCA2*	3,276	1,919	0.98	0.91 to 1.06	0.64
**10p15 - rs2380205**					
*BRCA1*	4,955	2,764	1.02	0.95 to 1.08	0.64
*BRCA2*	3,092	1,884	1.00	0.93 to 1.08	0.92
**11q13 - rs614367**					
*BRCA1*	6,177	3,144	1.01	0.94 to 1.10	0.73
*BRCA2*	3,310	1,944	0.99	0.89 to 1.10	0.88
**12q24 - rs1292011**					
*BRCA1*	3,773	1,798	1.04	0.97 to 1.12	0.29
*BRCA2*	2,345	1,220	0.96	0.88 to 1.06	0.41
**PTHLH - rs10771399**					
*BRCA1*	6,194	3,152	0.85	0.77 to 0.93	5.8 × 10^-4^
*BRCA2*	3,317	1,944	0.89	0.78 to 1.00	0.06
**9q31.2 - rs865686**					
*BRCA1*	6,196	3,149	1.01	0.95 to 1.07	0.72
*BRCA2*	3,315	1,946	0.94	0.87 to 1.02	0.15
***BRCA1 *analysis by mutation class**					
**CDK2NA/B -rs1011970**					
Class1	4,040	3,843	1.01	0.94 to 1.10	0.72
Class2	1,771	1,958	1.03	0.91 to 1.16	0.66
**ZNF365 - rs10995190**					
Class1	4,058	3,844	.99	0.92 to 1.07	0.80
Class2	1,774	1,957	0.97	0.86 to 1.09	0.59
**ZMIZ1 - rs704010**					
Class1	3,998	3,787	1.04	0.98 to 1.10	0.22
Class2	1,767	1,936	1.01	0.92 to 1.11	0.85
**10p15 - rs2380205**					
Class1	3,664	3,538	1.01	0.95 to 1.07	0.82
Class2	931	1,263	1.03	0.91 to 1.15	0.67
**11q13 - rs614367**					
Class1	4,024	3,833	1.10	1.02 to 1.19	0.02
Class2	1,764	1,948	0.94	0.84 to 1.06	0.32
**12q24 - rs1292011**					
Class1	2,812	2,521	0.99	0.92 to 1.06	0.71
Class2	642	797	0.97	0.84 to 1.12	0.68
**PTHLH - rs10771399**					
Class1	4,035	3,841	0.82	0.74 to 0.90	3.1 × 10^-5^
Class2	1,770	1,953	1.00	0.87 to 1.15	0.99
**9q31.2 - rs865686**					
Class1	4,038	3,840	0.98	0.92 to 1.04	0.48
Class2	1,769	1,957	1.03	0.94 to 1.14	0.49

HR, hazard ratio

We found no evidence of association between breast cancer risk for *BRCA1 *mutation carriers and any of the other SNPs under the trend models (*P*-trend > 0.15). There was, however, some suggestion of an association under the genotype specific model for rs865686 (2df *P *= 0.06, Table 2), reflecting a lower HR for heterozygous carriers than for either homozygote genotype. There was marginal evidence of heterogeneity in the HRs across countries for rs704010 and rs865686 (*P*-het = 0.04 for both), but examination of the forest plots revealed that in each case this was mainly due to a single study/country of relatively small sample size, with the majority of the HR estimates being close to 1 (Additional file 1 Supplementary Figure 1). There was no evidence that the HRs varied by age for any of the SNPs (*P *> 0.08 for all).

We further evaluated the SNP associations with breast and ovarian cancer risk simultaneously (Table 4). The associations with breast cancer risk remained essentially unchanged in the competing risk analysis, with only the *PTHLH *SNP rs10771399 being significantly associated with breast cancer risk. There was some suggestion of a possible association between this SNP and ovarian cancer risk for *BRCA1 *mutation carriers with risk in the opposite direction (HR for ovarian cancer = 1.14, 95% CI: 1.00 to 1.30, *P*-trend = 0.06) especially among rare homozygotes (ovarian cancer HR for GG = 1.67, 95% CI: 1.05 to 2.64, *P*-homozygotes = 0.03). This analysis also provided some weak evidence for an association between SNP rs614367 at 11q13 and ovarian cancer risk for *BRCA1 *mutation carriers under the genotype-specific model (2df *P*-value = 0.03). There was no evidence that any of the other SNPs are associated with ovarian cancer risk for *BRCA1 *mutation carriers.

**Table 4:** Competing risk analysis*.

					Breast cancer			Ovarian cancer		
		Unaffected N (%)	Breast cancer N (%)	Ovarian cancer N (%)	HR	95% CI	*P*-value	HR	95% CI	*P*-value
**CDK2NA/B - rs1011970**										
*BRCA1*	GG	3,328 (69.6)	4,424 (69.9)	1,026 (70.1)	1			1		
	GT	1,309 (27.4)	1,710 (27.0)	398 (27.2)	1.03	0.95 to 1.11		1.10	0.95 to 1.26	
	TT	142 (3.0)	194 (3.1)	39 (2.7)	1.09	0.88 to 1.35		0.90	0.61 to 1.32	
	2-df test						0.57			0.35
	per allele				1.04	0.91 to 1.11	0.30	1.04	0.93 to 1.17	0.48
*BRCA2*	GG	1,972 (68.6)	2578 (67.9)	315 (69.5)	1			1		
	GT	815 (28.4)	1097 (28.9)	129 (28.5)	1.09	0.98 to 1.21		1.07	0.85 to 1.35	
	TT	86 (3.0)	122 (3.2)	9 (2.0)	1.19	0.91 to 1.57		0.84	0.40 to 1.77	
	2-df test						0.15			0.74
	per allele				**1.09**	**1.00 to 1.19**	**0.05**	1.03	0.84 to 1.25	0.81
**ZNF365 - rs10995190**										
*BRCA1*	GG	3,408 (71.3)	4,523 (71.5)	1,019 (69.6)	1			1		
	GA	1,258 (26.3)	1,650 (26.1)	410 (28.0)	1.00	0.93 to 1.08		1.12	0.98 to 1.28	
	AA	113 (2.4)	155 (2.5)	35 (2.4)	0.96	0.78 to 1.20		0.90	0.61 to 1.33	
	2-df test						0.94			0.23
	per allele				0.99	0.93 to 1.06	0.88	1.06	0.95 to 1.19	0.32
*BRCA2*	GG	2,033 (70.4)	2,795 (73.7)	318 (70.2)	1			1		
	GA	794 (27.7)	920 (24.3)	122 (26.9)	**0.86**	**0.78 to 0.96**		0.99	0.78 to 1.25	
	AA	55 (1.9)	79 (2.1)	13 (2.9)	1.03	0.74 to 1.43		1.58	0.83 to 3.03	
	2-df test						**0.02**			0.37
	per allele				**0.90**	**0.82 to 0.99**	**0.03**	1.06	0.87 to 1.31	0.55
**ZMIZ1 - rs704010**										
*BRCA1*	CC	1,904 (40.2)	2,493 (40.0)	583 (40.1)	1			1		
	CT	2,172 (45.9)	2,871 (46.0)	665 (45.7)	1.02	0.95 to 1.10		1.01	0.89 to 1.15	
	TT	660 (13.9)	877 (14.1)	206 (14.2)	1.02	0.92 to 1.14		1.01	0.84 to 1.22	
	2-df test						0.67			0.99
	per allele				1.01	0.97 to 1.07	0.58	1.01	0.92 to 1.10	0.90
*BRCA2*	CC	1,109 (39.0)	1,439 (38.4)	181 (40.1)	1			1		
	CT	1,306 (46.0)	1,774 (47.3)	192 (43.4)	1.06	0.96 to 1.17		0.96	0.76 to 1.20	
	TT	426 (15.0)	538 (14.3)	69 (15.6)	1.00	0.86 to 1.15		1.05	0.77 to 1.43	
	2-df test						0.46			0.81
	per allele				1.01	0.95 to 1.08	0.72	1.01	0.87 to 1.18	0.90
**10p15 - rs2380205**										
*BRCA1*	CC	1,183 (32.2)	1,698 (32.1)	438 (33.2)	1			1		
	CT	1,796 (48.9)	2,605 (49.3)	634 (48.1)	0.99	0.91 to 1.08		0.91	0.79 to 1.05	
	TT	696 (18.9)	981 (18.6)	246 (18.7)	1.00	0.90 to 1.12		0.94	0.78 to 1.14	
	2-df test						0.96			0.45
	per allele				1.00	0.95 to 1.06	0.98	0.96	0.87 to 1.06	0.40
*BRCA2*	CC	872 (32.6)	1,161 (31.8)	143 (33.7)	1			1		
	CT	1,321 (49.4)	1,812 (49.6)	199 (46.9)	1.04	0.94 to 1.16		0.94	0.74 to 1.19	
	TT	481 (18.0)	678 (18.6)	82 (19.3)	1.03	0.90 to 1.18		1.01	0.75 to 1.38	
	2-df test						0.74			0.82
	per allele				1.02	0.95 to 1.09	0.61	1.00	0.85 to 1.16	0.98
**11q13 - rs614367**										
*BRCA1*	CC	3,439 (72.3)	4,547 (72.1)	1,111 (76.2)	1			1		
	CT	1,212 (25.5)	1,606 (25.5)	311 (21.3)	1.02	0.94 to 1.11		**0.83**	**0.72 to 0.96**	
	TT	109 (2.3)	154 (2.4)	37 (2.5)	1.12	0.91 to 1.39		1.20	0.81 to 1.76	
	2-df test						0.52			**0.03**
	per allele				1.03	0.91 to 1.10	0.35	0.91	0.80 to 1.03	0.13
*BRCA2*	CC	2,106 (73.5)	2,716 (71.9)	333 (73.8)	1			1		
	CT	693 (24.2)	981 (26.0)	108 (24.0)	1.05	0.95 to 1.16		0.95	0.74 to 1.21	
	TT	66 (2.3)	83 (2.2)	10 (2.2)	0.96	0.71 to 1.28		0.87	0.46 to 1.63	
	2-df test						0.62			0.84
	per allele				1.03	0.94 to 1.12	0.56	0.94	0.77 to 1.15	0.56
**12q24 - rs1292011**										
*BRCA1*	AA	997 (34.1)	1,321 (35.4)	305 (34.9)	1			1		
	AG	1,406 (48.2)	1,765 (47.3)	429 (49.1)	1.00	0.90 to 1.10		1.11	0.93 to 1.31	
	GG	517 (17.7)	645 (17.3)	140 (16.0)	1.01	0.89 to 1.15		0.98	0.78 to 1.24	
	2-df test						0.97			0.39
	per allele				1.00	0.94 to 1.07	0.91	1.01	0.91 to 1.13	0.82
*BRCA2*	AA	715 (35.0)	907 (35.9)	110 (36.5)	1			1		
	AG	961 (47.0)	1222 (48.4)	137 (45.5)	1.02	0.90 to 1.15		0.94	0.71 to 1.25	
	GG	370 (18.1)	396 (15.7)	54 (17.9)	**0.83**	**0.70 to 0.97**		0.89	0.61 to 1.28	
	2-df test						**0.03**			0.80
	per allele				0.93	0.86 to 1.00	0.07	0.94	0.78 to 1.13	0.51
**PTHLH - rs10771399**										
*BRCA1*	AA	3,810 (79.8)	5,179 (81.9)	1,145 (78.4)	1			1		
	AG	909 (19.0)	1078 (17.1)	289 (19.8)	0.89	0.81 to 0.97		1.09	0.93 to 1.26	
	GG	56 (1.2)	65 (1.0)	27 (1.9)	**0.86**	**0.63 to 1.16**		**1.67**	**1.05 to 2.64**	
	2-df test						**0.02**			0.06
	per allele				**0.90**	**0.83 to 0.97**	**6.4 × 10^-3^**	1.14	1.00 to 1.30	0.06
*BRCA2*	AA	2,289 (79.7)	3,076 (81.2)	369 (81.5)	1			1		
	AG	545 (19.0)	678 (17.9)	76 (16.8)	0.94	0.84 to 1.06		0.88	0.67 to 1.16	
	GG	37 (1.3)	34 (0.9)	8 (1.8)	0.79	0.49 to 1.26		1.48	0.63 to 3.46	
	2-df test						0.38			0.43
	per allele				0.93	0.84 to 1.04	0.19	0.96	0.75 to 1.23	0.75
**9q31.2 - rs865686**										
*BRCA1*	TT	1,935 (40.5)	2,621 (41.5)	605 (41.3)	1			1		
	TG	2,206 (46.2)	2,825 (44.7)	690 (47.1)	0.94	0.88 to 1.01		0.99	0.87 to 1.12	
	GG	633 (13.3)	877 (13.9)	169 (11.5)	1.03	0.93 to 1.15		0.85	0.70 to 1.03	
	2-df test						0.12			0.23
	per allele				1.00	0.95 to 1.05	0.88	0.94	0.86 to 1.03	0.17
*BRCA2*	TT	1,103 (38.4)	1,576 (41.6)	179 (39.6)	1			1		
	TG	1400 (48.8)	1712 (45.2)	215 (47.6)	**0.85**	**0.77 to 0.94**		0.91	0.73 to 1.14	
	GG	367 (12.8)	501 (13.2)	58 (12.8)	0.97	0.84 to 1.12		0.98	0.71 to 1.35	
	2-df test						**4.6 × 10^-3^**			0.70
	per allele				0.94	0.88 to 1.01	0.10	0.97	0.83 to 1.13	0.67

Associations with breast and ovarian cancer risk for *BRCA1 *and *BRCA2 *carriers.

*Censoring process described in the methods

HR, hazard ratio

### Associations with cancer risk for BRCA2 mutation carriers

There was evidence of association with breast cancer risk for *BRCA2 *mutation carriers for four SNPs (Table 2). The minor allele of rs10995190 in *ZNF365 *was associated with a reduced risk of breast cancer, where each copy of allele "A" was estimated to confer a HR of 0.90 (95% CI: 0.82 to 0.98, *P*-trend = 0.015). There was also some marginal evidence that the minor allele of rs1011970 near *CDKN2A/CDKN2B *was associated with increased breast cancer risk (HR = 1.09, 95% CI: 1.00 to 1.18, *P*-trend = 0.048). None of the other polymorphisms was associated with breast cancer risk for *BRCA2 *mutation carriers under the multiplicative model. However, SNPs rs865686 and rs1292011 were associated with risk under the genotype specific model (2df-*P *= 0.007 and 0.03 respectively, Table 2). There was some evidence of heterogeneity in the HRs across countries for rs1011970 (*P*-het = 0.005). This appeared to be mainly due to the USA stratum. The heterogeneity was no longer significant after removal of that stratum (*P*-het = 0.42) and the HR estimate for the association with breast cancer risk increased to 1.20 (95% CI: 1.09 to 1.32, *P*-trend = 1 × 10^-4^). There was no heterogeneity for any of the other SNPs (*P*-het > 0.12 for all, Additional file 1 Supplementary Figure 2). The HR estimates for the four associated SNPs were similar when long-term survivors were excluded from the analysis (Table 3). Consistent with the results of the main analysis, rs10995190 in *ZNF365 *and rs1011970 near *CDKN2A/CDKN2B *provided marginal evidence of association using the trend-test statistic (*P*-trend = 0.05 for both) and SNPs rs865686 was associated with breast cancer risk under the genotype specific model (2df-*P *= 0.03). SNP rs1292011 was not associated with breast cancer risk in this analysis. A somewhat smaller HR estimate was obtained for the *PTHLH *SNP rs10771399 compared to the main analysis (per-allele HR = 0.89, 95% CI: 0.78 to 1.00, *P*-trend = 0.06). The attenuation of the association in the overall analysis could have occurred if the SNP is also associated with prognosis. However, the difference in the HRs was small. The results for the remaining SNPs were similar and non-significant. None of SNPs were associated with ovarian cancer risk for *BRCA2 *mutation carriers (Table 4).

### Associations by tumour ER-status

Table 5 summarises the associations of the eight SNPs with breast cancer ER status in *BRCA1 *and *BRCA2 *mutation carriers. Only the *PTHLH *SNP rs10771399 was associated with ER-negative breast cancer for *BRCA1 *mutation carriers (ER-negative HR = 0.81, 95% CI: 0.74 to 0.90, *P*-trend = 3.8 × 10^-5^). There was also marginal evidence that SNP rs704010 near *ZMIZ1 *was associated with ER-positive breast cancer for *BRCA1 *mutation carriers (ER-positive HR = 1.12, 95% CI: 1.00 to 1.26, *P*-trend = 0.046). However, the associations between ER-negative and ER-positive breast cancer among *BRCA1 *mutation carriers were only significantly different for SNP rs1292011 at 12q24 (*P*-heterogeneity = 0.045).

**Table 5:** Associations with estrogen receptor-positive and estrogen receptor-negative breast cancer risk for *BRCA1 *and *BRCA2 *carriers.

					ER-positive			ER-negative			
	Unaffected N	ER-positive N	ER-negative N	ER status unknown N	HR	95% CI	*P*-value	HR	95% CI	*P*-value	P-dif
**CDK2NA/B - rs1011970**											
*BRCA1*	4,893	559	1,888	2,841	0.95	0.81 to 1.12	0.56	1.03	0.95 to 1.11	0.47	0.41
*BRCA2*	2,928	1,372	424	1,649	**1.10**	**1.00 to 1.22**	**0.05**	1.15	0.96 to 1.37	0.12	0.70
**ZNF365 - rs10995190**											
*BRCA1*	4,895	559	1,887	2,843	0.88	0.74 to 1.04	0.14	1.01	0.94 to 1.10	0.75	0.16
*BRCA2*	2,927	1,370	406	1,648	**0.89**	**0.80 to 1.00**	**0.043**	0.87	0.71 to 1.07	0.19	0.84
**ZMIZ1 - rs704010**											
*BRCA1*	4,842	548	1,846	2,811	**1.12**	**1.00 to 1.26**	**0.046**	1.00	0.94 to 1.06	0.91	0.08
*BRCA2*	2,887	1,347	401	1,636	1.01	0.93 to 1.09	0.91	1.00	0.87 to 1.14	0.95	0.91
**10p15 - rs2380205**											
*BRCA1*	4,465	540	1,812	2,513	0.90	0.80 to 1.01	0.08	1.02	0.96 to 1.09	0.46	0.06
*BRCA2*	2,701	1,341	396	1,543	1.02	0.95 to 1.10	0.60	0.94	0.82 to 1.08	0.39	0.31
**11q13 - rs614367**											
*BRCA1*	4,879	557	1,886	2,832	1.09	0.93 to 1.29	0.30	1.04	0.96 to 1.12	0.40	0.59
*BRCA2*	2,921	1,365	405	1,639	1.06	0.96 to 1.17	0.26	0.84	0.69 to 1.04	0.10	0.05
**12q24 - rs1292011**											
*BRCA1*	3,429	308	1,043	2,031	0.87	0.74 to 1.02	0.09	1.05	0.98 to 1.13	0.12	**0.046**
*BRCA2*	2,065	813	239	1,170	0.95	0.86 to 1.04	0.28	0.98	0.82 to 1.16	0.78	0.79
**PTHLH - rs10771399**											
*BRCA1*	4,889	557	1,887	2,842	0.94	0.78 to 1.13	0.52	**0.81**	**0.74 to 0.90**	**3.8 × 10^-5^**	0.20
*BRCA2*	2,926	1,366	406	1,648	0.97	0.86 to 1.10	0.68	**0.78**	**0.62 to 1.00**	**0.049**	0.12
**9q31.2 - rs865686**											
*BRCA1*	4,892	559	1,888	2,836	0.92	0.81 to 1.03	0.15	1.01	0.95 to 1.08	0.68	0.16
*BRCA2*	2,924	1,370	405	1,645	**0.91**	**0.84 to 0.99**	**0.028**	1.07	0.92 to 1.25	0.40	0.08

ER, estrogen receptor; HR, hazard ratio

Despite the small number of *BRCA2 *ER-negative breast cancers, there was a suggestion that the minor allele of the *PTHLH *SNP rs10771399 is protective for ER-negative breast cancer for *BRCA2 *mutation carriers (HR for ER-negative = 0.78, 95% CI: 0.62 to 1.00, *P*-trend = 0.049), but there was no association with ER-positive breast cancer. There was evidence that SNPs rs10995190 near *ZNF365*, rs865686 at 9q31.2 and rs1011970 near *CDKN2A/B *are associated with ER-positive breast cancer for *BRCA2 *mutation carriers (*P*-trend = 0.043, 0.028 and 0.05 respectively). However, the HR estimates were not significantly different from those for ER-negative breast cancer.

## Discussion

We have investigated eight novel breast cancer susceptibility loci identified through breast cancer GWAS [15-17] for their associations with breast cancer risk for *BRCA1 *and *BRCA2 *mutation carriers using data from the CIMBA. The estimated per-allele ORs associated with the minor allele of each SNP from the population-based studies varied from 0.85 to 1.15, and only four of the eight SNPs had ORs of less than 0.90 or greater than 1.10 (rs10995190, rs614367, rs865686 and rs10771399) [15-17]. For *BRCA1 *mutation carriers, only SNP rs10771399 at 12p11 was associated with the overall risk of breast cancer, whereas SNPs rs10995190 at 10q21, rs1011970 at 9p21, rs865686 at 9q31.2 and rs1292011 at 12q24 were associated with breast cancer risk for *BRCA2 *mutation carriers. The magnitude of the estimated HRs for all these SNPs were consistent with the OR estimates for the risk of breast cancer in the general population. The power to detect associations with SNPs conferring relative risks in the range of 0.90 to 1.10 was limited by our sample size, particularly among *BRCA2 *mutation carriers [29].

Based on the HR estimates and associated 95% confidence intervals, given our sample size of *BRCA1 *mutation carriers, it is unlikely that the relative risks for overall *BRCA1 *breast cancer risk are of similar magnitude to those estimated in the general population for SNPs rs10995190 at 10q21 (estimated odds ratio (OR) from the replication stage of the GWAS = 0.76), rs2380205 at 10p15 (OR = 0.94), rs614367 at 11q13 (OR = 1.15), rs1292011 at 12q24 (OR = 0.92) and rs865686 at 9q31.2 (OR = 0.89), since the 95% confidence intervals for the HRs do not include the estimated OR from the population-based studies. Similarly, the HRs for *BRCA2 *breast cancer risk exclude the ORs from the general population for SNPs rs2380205 at 10p15 and rs614367 at 11q13. Taken together, these findings suggest that SNPs rs2380205 at 10p15 and rs614367 at 11q13 do not modify breast cancer risk in either *BRCA1 *or *BRCA2 *mutation carriers. A replication study by BCAC, involving close to 50,000 breast cancer cases and 50,000 controls, found only weak evidence for association of rs2380205 at 10p15 with breast cancer risk in the general population [Lambrechts and Easton personal communication, manuscript submitted] suggesting that the original finding (OR = 0.94, *P *= 5 × 10^-7 ^[15]) may have been a false positive. If this were true, the absence of an association in carriers would be expected. The lack of evidence for an association with the 11q13 SNP rs614367 with *BRCA1 *and *BRCA2 *breast cancer risk is more surprising since the association in the general population is relatively strong and consistently replicated (OR 1.21, 95% CI 1.17 to 1.25 in the recent BCAC analysis [Lambrechts and Easton personal communication, manuscript submitted]). The association in the general population appears to be restricted to ER-positive disease, which would explain the lack of association for *BRCA1 *carriers but not *BRCA2 *carriers. This is perhaps the clearest evidence so far of a departure from a multiplicative interaction between a common susceptibility locus and a *BRCA2 *mutation on the risk of developing breast cancer. The lack of an association in *BRCA1 *carriers for rs1292011 and rs865686 is also consistent with the observation that these associations are stronger for ER-positive disease in the general population [16]. The absence of an association for *ZNF365 *rs10995190 in *BRCA1 *carriers is more surprising since this association appears to be unrelated to ER status in the general population [Lambrechts and Easton personal communication, manuscript submitted] [30].

Of the eight SNPs investigated, the strongest association was found between SNP rs10771399 at 12p11 and breast cancer risk for *BRCA1 *mutation carriers. Other loci previously found to be associated with *BRCA1 *breast cancer risk include the 19p13 and 6q25.1 loci [6,9], *TOX3 *and *CASP8 *[3,5,7]. Analysis by tumour ER-status revealed that rs10771399 at 12p11 has a stronger association with ER-negative than ER-positive breast cancer for both *BRCA1 *and *BRCA2 *mutation carriers. The ER-specific HRs were similar for both genes, suggesting that this SNP is primarily associated with ER-negative breast cancer, although results from the general population suggested similar ORs for ER-positive and ER-negative breast cancer (0.87 for ER-positive disease, 0.85 for ER-negative disease [16]). Interestingly, the association among *BRCA1 *mutation carriers was restricted to those carrying mutations proven or predicted to lead to absence of protein expression (Class 1) with no evidence for an association in carriers of *BRCA1 *mutations likely to generate stable mutant proteins (Class 2) (*P*-diff = 0.03). This observation suggests that the modifying effect of SNP rs10771399 at 12p11 might be attenuated for tumours that retain residual *BRCA1 *function or that retain the capacity to bind to some of its partners. rs10771399 lies in a region at 12p11 that contains *PTHLH *(parathyroid hormone-like hormone isoform 1, also known as *PTHRP - *parathyroid hormone-related protein) and *CCDC91. PTHLH *is a plausible candidate cancer susceptibility gene. It encodes a protein that regulates endochondral bone development and epithelial-mesenchymal interactions during the formation of the mammary glands. The receptor of this hormone, PTHR1, is responsible for most cases of humoral hypercalcemia of malignancy [31]. It is produced by various types of carcinomas [32], and is an important factor in the development of bone metastasis [33].

We found that SNP rs10995190 *ZNF365 *is associated with *BRCA2 *breast cancer risk. A different SNP (rs16917302) in *ZNF365*, which is only weakly correlated with rs10995190 (pairwise r^2 ^is approximately 0.10 in the present sample) was previously identified via a GWAS of breast cancer in *BRCA2 *mutation carriers [10]. These results suggest that there could be a causal associated variant correlated with both rs10995190 and rs16917302, or alternatively more than one causal disease variant in this locus. SNP rs10995190 has also recently been found to be associated with mammographic density in the general population [34]. Previous studies found that mammographic density modifies breast cancer risk for *BRCA2 *mutation carriers [35], raising the possibility that this locus modifies breast cancer risk for *BRCA2 *mutation carriers through its influence on mammographic density. However, mammographic density has also been shown to modify the breast cancer risk for *BRCA1 *carriers, which also makes the absence of association for rs10995190 in *BRCA1 *carriers somewhat surprising. Mammographic density data are not available in the CIMBA sample to test this hypothesis explicitly.

There was no evidence of association with ovarian cancer risk for *BRCA1 *or *BRCA2 *mutation carriers for any of the SNPs, with the exception of some weak evidence for SNPs rs10771399 and rs614367 for *BRCA1 *carriers. This is not surprising, since all SNPs were selected on the basis of prior evidence of association with breast cancer risk in the general population and none of these SNPs have so far been found to be associated with ovarian cancer in general population through the ongoing GWAS [36-38].

## Conclusions

The per-allele HRs estimated for each of the associated loci in the present report are modest, and in isolation would have only a small impact on the absolute risks of developing breast cancer. However, we have shown previously that modifier SNPs in combination can result in large differences in the absolute risk of developing breast cancer for carriers at the extreme percentiles of the combined SNP distribution [5,39]. Furthermore, the causal variants underlying these loci may confer larger relative risks. Considering all reported modifying loci by the CIMBA consortium, there are now six loci in total that are associated with breast cancer risk for *BRCA1 *mutation carriers (19p13, 6q25.1, 12p11, *TOX3*, 2q35 and C*ASP8*) and 13 loci which are known to be associated with *BRCA2 *breast cancer risk (*FGFR2, TOX3, MAP3K1, LSP1*, 2q35, *SLC4A7*, 5p12, 1p11.2, *ZNF365, CDKN2A/B*, 9q31.2, 12q24 and *RAD51*). Ongoing GWAS in *BRCA1 *and *BRCA2 *mutation carriers and in the general population are likely to identify further modifier loci and taken together, they may lead to more accurate risk predictions in mutation carriers with implications for clinical management, and to a better understanding of the biology of tumour development in mutation carriers.

## Abbreviations

BCAC: Breast Cancer Association Consortium; CIMBA: Consortium of Investigators of Modifiers of *BRCA1/2; *ER: estrogen receptor; GWAS: genome-wide association studies; HR: hazard ratio; HWE: Hardy-Weinberg equilibrium; NMD: nonsense-mediated mRNA decay; OR: odds ratio; SNPs: single-nucleotide polymorphism.

## Competing interests

The authors declare that they have no competing interests.

## Authors' contributions

ACA, KBK and DFE wrote the manuscript. KBK performed the statistical analysis. ACA supervised the statistical analysis and data management. ACA, GCT and DFE developed the study design. LM and DB are the CIMBA database managers. AL wrote computer programs for the analysis. SH and OMS reviewed, recoded and classified the BRCA1 and BRCA2 mutations in CIMBA. GCT initiated and coordinates CIMBA. PS, JB, XC and YCD performed the genotyping. AJ, SLN, GCT and JS supervised the genotyping of samples. MAC, NL, KH, AL, BA, RR, PK, KN, SD, TR, AJ, JL, KJ, KD, EZ, AO, MD, RA, JB, UH, FBH, TAvO, SV, HEJMH, JW, EBGG, MJL, MK, JMC, MGEMA, JCO, SP, DF, SDE, RP, EF, DGE, FL, CJ, RE, JA, RD, TC, JC, JP, FD, CB, SH, PJM, LW, MTR, AD, HD, AKG, BB, DS, CH, BB, AdP, SM, AC, ML, BBdeP, OC, HS, MF, FP, SFF, IM, SB, MD, AM, MBT, JLH, EMJ, MS, DG, CFS, AFR, MKT, DGK, TvOH, FCN, RBB, MG, TK, VJ, ADC, KO, MP, JK, DC, JH, JB, JF, AET, MM, CO, EI, CI, LT, IB, CL, AT, JDV, SAG, KO, JG, BYK, EO, SHT, PAG, MSB, CMD, EJvR, OD, AK, RKS, BW, CE, AM, ND, NA, SH, DN, SPA, DG, RVM, HD, AG, CS, KK, BF, DS, TC, MdlH, HN, TAM, BL, ABS, SLN, YCD, XW, ZF, VSP, NML, PR, MHG, JTL, ILA, HO, AMM, GG, MT, AMG, UBJ, ABS, TAK, GCT and FJC acquired phenotypic data and DNA samples or designed the centre-specific studies. All authors read and approved the final manuscript for publication.

## Supplementary Material

Additional file 1 **Supplementary tables and figures**. Table S1 List of local ethics committees that granted approval for the access and use of the data in current study. Supplementary figure 1 Forest plot of the country-specific per-allele HR estimates for breast cancer for *BRCA1 *mutation carriers. Supplementary figure 2 Forest plot of the country-specific per-allele HR estimates for breast cancer for *BRCA2 *mutation carriers. Click here for file
