# Supplementary material for: Common variants at 12p11, 12q24, 9p21, 9q31.2 and in ZNF365 are associated with breast cancer risk for BRCA1 and/or BRCA2 mutation carriers
Source: Breast Cancer Res. 2012 Feb 20;14(1):R33. doi: 10.1186/bcr3121 (PMC3496151; doi:10.1186/bcr3121)

| **Suplementary Table 1:**  List of local ethics committees that granted approval for the access and use of the data in current study | | |
| --- | --- | --- |
| **Study** | **Country** | **Committee approval** |
| Breast Cancer Family Registry (BCFR) | USA | Institutional Review Board University of Utah |
| (BCFR - addtional) | Australia | The University of Melbnourne Health Sciences Human Ethics Sub-Committee |
| (BCFR - addtional) | USA | Columbia University Medical Center Institutional Review Board |
| (BCFR - addtional) | USA | Northern Californa Cancer Center Institutional Review Board |
| (BCFR - addtional) | Canada | University Health Network Research Ethics Board |
| (BCFR - addtional) | Canada | Mount Sinai Hospital Research Ethics Board |
| Baltic Familial Breast and Ovarian Cancer Consortium (BFBOCC) | Latvia, Lithuania | Centrālā medicīnas ētikas Komiteja |
| BRCA-gene mutations and beast cancer in South African women (BMBSA) | South Africa | Univ. of Pretoria and Pretoria Academic Hospitals Ethics Committee |
| Copenhagen Breast Cancer Study (CBCS) | Denmark | De Videnskabsetiske Komiteer I Region Hovedsladen |
| Spanish National Cancer Centre (CNIO) | Spain, Greece | Instituto de Salud Carlos III Comité de Bioética y Bienestar Animal |
| CONsorzio Studi ITaliani sui Tumori Ereditari Alla Mammella (CONSIT TEAM) | Italy | Comitato Etico Indipendente della Fondazione IRCCS "Istituto Nazionale dei Tumori" |
| Deutsches Krebsforschungszentrum (DKFZ) | Germany | Ethik-Kommission des Klinikums der Universität |
| (DKFZ - addtional) | Columbia | Hospital Universitario de San Ignacio Comité de Investigaciones y Etica |
| (DKFZ - addtional) | Pakistan | Shaukat Khanum Memorial Cancer Hospital and Research Centre Institutional Review Board |
| HEreditary Breast and Ovarian study Netherlands (HEBON) | The Netherlands | Protocol Toetsingscommissie van het Nederlands Kanker Instituut/Antoni van Leeuwenhoek Ziekenhuis |
| Epidemiological study of BRCA1 and BRCA2 mutation carriers (EMBRACE) | UK and EIRE | Anglia & Oxford MREC |
| Fox Chase Cancer Center (FCCC) | USA | Institutional Review Board Fox Chase Cancer Center |
| German Consortium of Hereditary Breast and Ovarian Cancer (GC-HBOC) | Germany | Ethik-Kommission der Medizinischen Fakultät der Universät zu Köln |
| Genetic Modifiers of cancer risk in *BRCA1*/2 mutation carriers (GEMO) | France, USA | Comité consultatif sur le traitement de I'information en matière de recherche dans le domaine de la santé |
| Georgetown University (GEORGETOWN) | USA | MedStar Research Institute - Georgetown University Oncology Institutional Review Board |
| Gynecologic Oncology Group (GOG) | USA | National Cancer Institute - Cancer Prevention and Control Concept Review Committee |
| Hospital Clinico San Carlos (HCSC) | Spain | Comité Ético de Investigación Clínia Hospital Clínico San Carlos |
| Helsinki Breast Cancer Study (HEBCS) | Finland | Helsingin ja uudenmaan sairaanhoitopiiri (Helsinki University Central Hospital ethics committee) |
| Hungarian Breast and Ovarian Cancer Study (HUNBOCS) | Hungary | Institutional Review Board of the Hungarian National Institute of Oncology |
| Univeristy Hospital Vall d'Hebron (HVH) | Spain | The Hospital Universitario Vall d'Hebron Clinical Research Ethics Committee |
| Institut Català d'Oncologia (ICO) | Spain | Catalan Institute of Oncology Institutional Review Board |
| International Hereditary Cancer Centre (IHCC) | Poland | Komisji Bioetycznej Pomorskiej Akademii Medycznej (Pomeranian Medical University Bioethics Committee) |
| Iceland Landspitali - University Hospital (ILUH) | Iceland | Vísindasiđanefnd National Boethics Committee |
| Interdisciplinary Health Research International Team Breast Cancer Susceptibility (INHERIT) | Quebec -Canada | Comité d'éthique de la recherche du Centre Hospitalier Universitaire de Québec |
| Istituto Oncologico Veneto Hereditary Breast and Ovarian Cancer Study (IOVHBOCS) | Italy | Centro Oncologico Regionale Azienda Ospedale Di Padova Comitato Etico |
| Kathleen Cuningham Foundation Consortium for Research into Familial Breast Cancer (KCONFAB) | Australia | Peter MacCallum Cancer Centre Ethics Committee |
| (KCONFAB - additional) | Australia | Queensland Institute of Medical Research - Human Research Ethics Committee |
| Mayo Clinic (MAYO) | USA | Mayo Clinic Institutional Review Boards |
| Memorial Sloane Kettering Cancer Center (MSKCC) | USA | Memorial Sloan-Kettering Cancer Center IRB |
| (MSKCC - additional) | USA | Human Biospecimen Utilization Committee |
| General Hospital Vienna (MUV) | Austria | Ethikkommission der Medizinischen Universität Wien |
| National Cancer Institute (NCI) | USA | NIH Ethics Office |
| Ontario Cancer Genetics Network (OCGN) | Canada | Mount Sinai Hospital Research Ethics Board |
| The Ohio State University Comprehensive Cancer Centre (OSU-CCG) | USA | Cancer Institutional Review Board |
| Odense University Hospital (OUH) | Denmark | Den Videnskabsetiske Komité for Region Syddanmark |
| Swedish Breast Cancer Study (SWE-BRCA) | Sweden | Regionala Etikprövningsnämnden Stockholm |
| N.N. Petrov Institute of Oncology (NNPIO) | Russia | N.N. Petrov Institional Ethical Committee |
| University of California Irvine (UCI, now BRICOH) | USA | UC Irvine: Office of Research Administration Institutional Review Board |
| University of California Los Angeles (UCLA) | USA | UCLA Institutional Review Board |
| University of California San Francisco (UCSF) | USA | Committee on Human Research |
| UK and Gilda Radner Familial Ovarian Cancer Registries (UKGRFOCR) | UK | Cambridge Local Research Ethics Committee |
| (UKGRFOCR - additional) | USA | Roswell Park Cancer Institute IRB |
| University of Pennsylvania (UPENN) | USA | University of Pennsylvania Institutional Review Board |
| Women’s Cancer Research Institute (WCRI) | USA | Cedars-Sinai Institutional Review Board |

| **Supplementary Table 2**: Number of eligible *BRCA1* and *BRCA2* carriers by study group used in the primary analysis. | | | | |
| --- | --- | --- | --- | --- |
| **Study** | **Country^1^** | ***BRCA1*, N** | ***BRCA2*, N** | **Genotyping Centre^2^** |
| Breast Cancer Family Registry (BCFR) | USA, Canada, Australia | 481 | 328 | QB |
| Baltic Familial Breast Ovarian Cancer Consortium (BFBOCC) | Latvia, Lithuania | 115 | 0 | QB |
| BRCA-gene mutations and breast cancer in South African women (BMBSA) | South Africa | 67 | 152 | QB |
| Copenhagen Breast Cancer Study (CBCS) | Denmark | 194 | 76 | QB |
| Spanish National Cancer Centre (CNIO) | Spain, Greece | 169 | 206 | QB |
| CONsorzio Studi ITaliani sui Tumori Ereditari Alla Mammella (CONSIT TEAM) | Italy | 602 | 365 | QB |
| Deutsches Krebsforschungszentrum (DKFZ) | Germany | 66 | 27 | QB |
| HEreditary Breast and  Ovarian study Netherlands  (HEBON) | The Netherlands | 777 | 290 | QIMR |
| Epidemiological study of BRCA1 and BRCA2 Mutation Carriers (EMBRACE) | UK and Eire | 1158 | 978 | QIMR |
| University of Kansas Medical Center (KUMC) | USA | 123 | 96 | QIMR |
| German Consortium of  Hereditary Breast and  Ovarian Cancer (GC-HBOC) | Germany | 1126 | 525 | QB |
| Genetic Modifiers of  cancer risk in *BRCA1*/2  mutation carriers (GEMO) | France, USA | 1083 | 627 | QB |
| Georgetown University (GEORGETOWN) | USA | 34 | 27 | QIMR |
| Gynecologic Oncology Group (GOG) | USA | 370 | 260 | QB |
| Hospital Clinico San Carlos (HCSC) | Spain | 133 | 122 | QB |
| Helsinki Breast Cancer Study (HEBCS) | Finland | 101 | 100 | QIMR |
| Hungarian Breast and Ovarian Cancer Study (HUNBOCS) | Hungary | 150 | 44 | QB |
| University Hospital Vall d'Hebron (HVH) | Spain | 52 | 61 | QB |
| Institut Català d'Oncologia (ICO) | Spain | 178 | 190 | QB |
| International Hereditary Cancer Centre (IHCC) | Poland | 1442 | 0 | PMU |
| Iceland Landspitali - University Hospital (ILUH) | Iceland | 0 | 170 | QIMR |
| Interdisciplinary Health  Research International  Team Breast Cancer  Susceptibility (INHERIT  BRCAs) and McGill University | Quebec - Canada | 51 | 61 | QB |
| Istituto Oncologico Veneto - Hereditary Breast Ovarian Cancer Study (IOVHBOCS) | Italy | 142 | 120 | QB |
| kConFab | Australia | 658 | 534 | QIMR |
| Beckman Research Institute City of Hope (BRICOH) | USA | 223 | 164 | COH |
| Mayo Clinic (MAYO) | USA | 268 | 188 | QIMR |
| Memorial Sloane Kettering Cancer Center (MSKCC) | USA | 264 | 194 | QB |
| Medical University Vienna | Austria | 306 | 143 | QIMR |
| National Cancer Institute (NCI) | USA | 154 | 65 | QB |
| Ontario Cancer Genetics Network (OCGN) | Canada | 211 | 168 | QB |
| Ohio State University Clinical Cancer Center (OSU CCG) | USA | 90 | 52 | QB |
| Odense University Hospital (OUH) | Denmark | 276 | 199 | QB |
| Pisa Breast Cancer Study (PBCS) | Italy | 95 | 63 | QIMR |
| Swedish Breast Cancer Study (SWE-BRCA) | Sweden | 536 | 167 | QIMR |
| N.N. Petrov Institute of Oncology (NNPIO) | Russia | 91 | 0 | QB |
| University of California Los Angeles (UCLA) | USA | 74 | 57 | COH |
| University of California San Francisco (UCSF) | USA | 43 | 27 | QB |
| UK and Gilda Radner Familial Ovarian Cancer Registries (UKGRFOCR) | UK/USA | 158 | 32 | QB |
| University of  Pennsylvania (UPENN) | USA | 365 | 178 | QIMR |
| Cedars-Sinai Medical Center (WCRI) | USA | 173 | 76 | QB |
|  |  |  |  |  |
| Total |  | 12599 | 7132 |  |
| 1: Country of the clinic at which carriers are recruited  2: Genotyping centres: QB=Cancer Genomics Laboratory, Centre Hospitalier Universitaire de Québec and Laval University,Canada; QIMR=Queensland Institute of Medical Research; PMU=Pomeranian Medical University, Szczecin Poland; COH=Beckman Research Institute of the City of Hope, Duarte, CA USA | | | | |

Supplementary figure 1: Forest plot of the country-specific per-allele HR estimates for breast cancer for *BRCA1* mutation carriers.

Supplementary figure 2: Forest plot of the country-specific per-allele HR estimates for breast cancer for *BRCA2* mutation carriers.


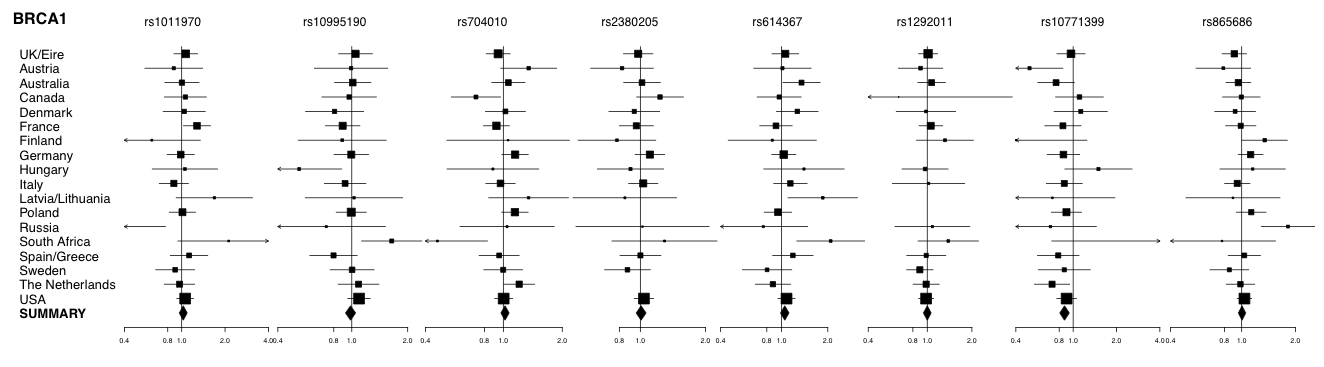

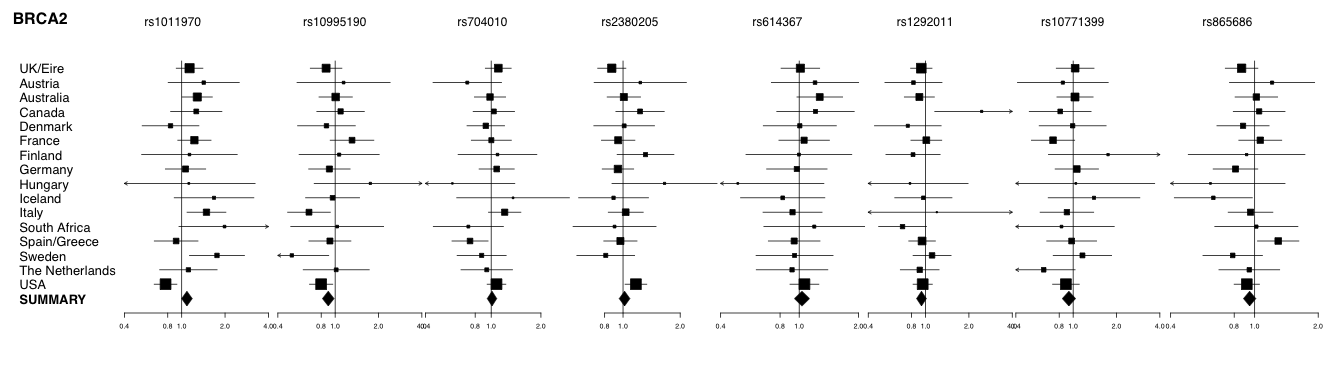

Supplement: Additional file 1 — Supplementary tables and figures. Table S1 List of local ethics committees that granted approval for the access and use of the data in current study. Supplementary figure 1 Forest plot of the country-specific per-allele HR estimates for breast cancer for BRCA1 mutation carriers. Supplementary figure 2 Forest plot of the country-specific per-allele HR estimates for breast cancer for BRCA2 mutation carriers. [file bcr3121-S1.DOCX]
